# Single-step cycle pulse operation of the label-free electrochemiluminescence immunosensor based on branched polypyrrole for carcinoembryonic antigen detection

**DOI:** 10.1038/srep24599

**Published:** 2016-04-19

**Authors:** Wenjuan Zhu, Qi Wang, Hongmin Ma, Xiaohui Lv, Dan Wu, Xu Sun, Bin Du, Qin Wei

**Affiliations:** 1 Key Laboratory of Chemical Sensing & Analysis in Universities of Shandong, School of Chemistry and Chemical Engineering, University of Jinan, Jinan 250022, P.R. China; 2 School of Material Science and Engineering, University of Jinan, Jinan 250022, P.R. China

## Abstract

A novel label-free electrochemiluminescence (ECL) immunosensor based on luminol functional-Au NPs@polypyrrole has been developed for the detection of carcinoembryonic antigen (CEA). In this work, polypyrrole prepared by chemical polymerization provided a large surface area to load amounts of gold nanoparticles (Au NPs). Au NPs could not only attach abundant luminol for the enhancement of ECL signal, but also provide a friendly microenvironment for the immobilization of antibodies. Moreover, 1-butylpyridinium tetrafluroborate ([BPy]BF_4_) were used to disperse luminol functional-Au NPs@polypyrrole nanocomposites, resulting in the film-formation of composites on the electrode, which could improve the stability of immunosensor. In particular, employment of single-step cycle pulse could limit the consecutive reaction between luminol and H_2_O_2_ efficiently, thus leading to stable and strong signals. The proposed method presents good ECL response for the detection of CEA allowing a wide linear range from 0.01 pg/mL to 10 ng/mL and a limit of detection as low as 3 fg/mL. The immunosensor would be a promising tool in the early diagnosis of CEA due to its high sensitivity, simplicity and cost-effective.

Cancer is the most common malignancy, which is one of the major causes of high mortality for human beings. The clinical diagnosis for cancer biomarkers at early stage will contribute to the cure of cancer and avoid a fatal outcome^1^,^2^,^3^. Carcinoembryonic antigen (CEA) is a kind of glycoprotein which is generated by tumor tissues in the human body. The level of CEA can respond for the presence or recurrence of colon tumors^4^ and breast tumors^5^, which also displays a substantial correlation with ovarian carcinoma^6^, cervical carcinoma^7^ and other cancers. Simultaneously, the CEA level in serum is also closely related to the growth state of the tumor, which can monitor the process of treatment or chances of recovery and evaluate curative effect. So the quantitative detection of CEA in serum will be valuable for clinical research and early diagnosis.

Up to now, a series of analysis strategies for the detection of CEA have been reported, such as amperometry^8^, capillary electrophoresis^9^, fluorometry^10^ and electrochemistry^11^. However, all of these techniques have some defects more or less, such as base current interference, time-consuming and sophisticated instrumentation. Currently, a widespread attention has been attracted by the development of the immunochemical method based on the antigen-antibody reaction^12^,^13^. Especially, electrochemiluminescence (ECL) technology is the most noteworthy due to the various advantages including high sensitivity, low background signal and fast sample analysis^14^,^15^,^16^. Across the ECL detection process, luminol is often adopted as an ECL reagent due to its low oxidation potential and high ECL efficiency, which is beneficial for the increment of accuracy and sensitivity. Meanwhile, to further enhance the chemiluminescence of luminol, metal nanoparticles were often applied as catalysts^17^,^18^. Considering the unique catalytic and electronic properties, gold nanoparticles (Au NPs) was selected to enhance the ECL of luminol-H_2_O_2_ system through the catalysis of Au NPs on the radical generation and electron-transfer processes during the luminol chemiluminescence reaction. The possible mechanism^19^ was described as follows. With the appearance of Au NPs, H_2_O_2_ might be broken up into double free radicals HO^•^ which could be stabilized by Au NPs^20^. The free radicals HO^•^ further reacted with luminol anion (L^−^) and hydrogen peroxide anion (H_2_O^−^) to from luminol radicals (L^−•^) and superoxide radical anion (O_2_^−•^). Then on the surface of Au NPs, L^−•^ and O_2_^−•^ underwent electron-transfer processes to generate the excited state [LO_2_^2−^]^*^. When [LO_2_^2−^]^*^ fell from the excited state to the ground state, light was detected. In this study, Au NPs could not only attach abundant luminol via an amide-forming reaction^21^ and enhance luminol chemiluminescence, but also provide a friendly microenvironment to conjugate with antibodies^22^. Moreover, the introduction of Au NPs with high conductivity is also beneficial to promote the electron transfer process, thus leading to the improvement of the sensitivity in the as fabricated biosensor.

In order to immobilize more Au NPs on the electrodes, polypyrrole (PPy) is adopted as an environmental binder material due to its great stability, large specific surface area and simple synthesis of free surfactants or templates^23^,^24^. Particularly, nanostructured PPy also played a vital role to increase the electrical conductivity of the biosensor, in that their long π-conjugated backbone facilitating the rapid electron transfer. These excellent properties attract great interest in the field of biosensor for the possible implementation of PPy on the constructing of electrode interfaces.

Herein, a promising label-free ECL immunosensor was fabricated, which was prepared by luminol-Au NPs@PPy as antibody carriers and sensing platform for the detection of CEA. The fabrication procedure of the immunosensor was shown in Fig. 1. PPy synthesized through a convenient synthetic route of chemical polymerization^25^ was used to immobilize Au NPs. Au NPs were used to combine with luminol and anti-CEA, and further to enhance the sensitivity of immunosensor. Considered of the excellent properties of ionic liquid, such as low toxicity, enhanced ionic conductivity, good chemical stability^26^, 1-butylpyridinium tetrafluroborate ([BPy]BF_4_) was adopted to disperse anti-CEA-luminol-Au NPs@PPy composites, which could not only result in the film-formation of composites for the immobilization on the electrode, but also improve the stability of immunosensor. Additionally, single-step cycle pulse was enforced to electrodes, causing that the fabricated sensing platform performed stable and strong ECL signals. The proposed label-free ECL immunosensor exhibited high sensitivity for CEA with a linear range from 0.01 pg/mL to 10 ng/mL and a low detection limit of 3 fg/mL. It would be promising and effective method for the recognition of target CEA.

## Results and Discussion

### Characterization of PPy, Au NPs@PPy and anti-CEA-luminol-Au NPs@PPy

The morphologies of PPy and Au NPs@PPy were characterized by SEM. As shown in Fig. 2A, it could be observed visually that PPy was branched structure, showing uniform shapes and smooth surface. In addition, it was clear in Fig. 2B that a large number of Au NPs have been modified successfully on the surface of PPy. To further prove the luminol, anti-CEA, Au NPs and PPy were incubated together, the correlative UV-vis spectrums (Fig. 2C) were investigated. The maximum absorption peak of the as-synthesized PPy (curve a) was showed at 296 nm. Obvious absorption peak of luminol (curve c) was observed at around 300 nm and 348 nm. The characteristic absorption peak Au NPs (curve b) and anti-CEA (curve d) was identified at around 519 nm and 285 nm respectively. Curve e was the absorption curve of anti-CEA-luminol-Au NPs@PPy. As Fig. 2C (curve e) showed, the characteristic absorption peak of the luminol was apparently broadened and the major absorption peak of Au NPs also appeared, which illustrated that PPy, luminol, anti-CEA and Au NPs were incubated well effectively.

Figure 2D shows the FT-IR spectrum of PPy. The broad peak at 3429 cm^−1^ is due to N-H stretching vibration of PPy ring^27^. The peak at around 1630 cm^−1^ depicts the aromatic C=C stretching of PPy^28^. The existing peaks at near 1563 cm^−1^ may attributed to the five-membered ring stretching of PPy^29^. Moreover, the peaks observed at 1195 and 1053 cm^−1^ are corresponding to C-N stretching and N-H out-of-plane bending of PPy. The peak corresponding to near 690 cm^−1^ is due to the aromatic out of plane bending vibration of PPy rings. The peak at 800–930 cm^−1^ may depict C-H in plane and out-of-plane deformations in pyrrole monomer^27^. These peaks further suggested that the preparation of PPy was successful.

### Characterization of the immunosensor

Electrochemical impedance spectroscopy (EIS) was used to monitor the stepwise assembly of the immunosensor taking place in the presence of iron ferrocyanides (Fe(CN)_6_^3−/4−^, 2.5 mM). It was an effective and convenient method to study the interfacial resistance of different modified electrodes without destruction by directly converting bioactivator into electrical signal^30^,^31^. The EIS measurements were carried out in the frequency range from 0.1 to 100 kHz. The EIS curves of the GCE at different modification states were shown in Fig. 3A. In detail, there was a small semicircle domain on the bare electrode, which attributed to the diffusion limited electrochemical process. When the electrode was immobilized with anti-CEA-luminol-Au NPs@PPy (curve c), its diameter of the semicircle domain markedly increased compared with the electrode modified with luminol-Au NPs@PPy (curve b) due to the obstruction of anti-CEA for the electron transfer from the electrode to the solution. This phenomenon confirmed that the anti-CEA was bonded to luminol-Au NPs@PPy successfully. After BSA was dropped onto the electrode (curve d), the charge transfer resistance value increased sharply, which was attributed to the fact that the BSA attached onto Au NPs or PPy via gold-amino bonds or physical absorption resulting the conductive support and counteracting the interfacial electron transfer^32^. After successful capture of CEA, the diameter of the semicircle increased (curve e). The increased charge transfer resistance caused by that the antigen-antibody complex was generated on the electrode through specific reaction, which blocked the electron transfer^33^. The results were consistent with the fact that the electrode was modified as expected. Simultaneously, the corresponding ECL responses of different modified electrodes were investigated (Fig. 3B). As expected, the ECL intensity of electrodes was obviously decreased with stepwise fabrication. It further proved that the label-free ECL immunosensor was successfully fabricated.

### Selection of electrochemical parameters

As far as we know ECL reaction is initiated by an electrochemical reaction at the electrode surface, the electrochemical parameters play very important roles in the ECL response. In this work, two different electrochemical techniques including cyclic voltammetry (Fig. 4A) and single-step cycle pulse (Fig. 4B) were investigated. The results showed that the ECL signals for the single-step cycle pulse were more stable and much stronger than that for cyclic voltammetry. The reasons might as follows. In the pattern of single-step cycle pulse, the diffusion layer on the surface of electrodes could be restored timely resulting in reproducible signals. Simultaneously, the electrode reaction with single-step cycle pulse could produce more photons than that for cyclic voltammetry with the same time, which causing stronger ECL signal.

Other electrochemical parameters were also explored, including initial potential, pulse potential, pulse period and pulse time. Figure 4C showed the effect of initial potential on the ECL emission. Interestingly, ECL emission increased with initial potential between −0.45 and −0.30 V. Further increased in initial potential resulted in a decrease in ECL emission. It could be attributed that the initial potential of −0.30 V provided a more favorable condition for the diffusion controlled reaction on the surface of the electrode. Figure 4D illustrated that pulse potential had great influence on ECL emission. With pulse potential at 0.5 V, the sensor produced a relatively maximal signal. When pulse potential was more than 0.5 V, ECL emission was attenuated and along with the poor stability due to the electro-oxidation of Au NPs on the surface of electrode. The result in Fig. 4E showed that a maximal ECL intensity was achieved at the pulse period value of 12 s. This was probably because it required a suitable time for the diffusion of H_2_O_2_ from electrolyte solution to electrode to contact with luminol. In the meantime, it was observed obviously in the Fig. 4F that there was a threshold of pulse time at 0.3 s for the generation of stable ECL signal. When the pulse time was over 0.3 s, ECL emission was decreased, which might be attributed to the fact that with a relatively long pulse time, the diffusion layer would become thicker on the surface of electrode and it was difficult to recover in the next pulse. Therefore, a pulse time of 0.3 s was adopted in the following experiments.

### Optimization of assay conditions

As is well-known, the ECL intensity of the luminol-H_2_O_2_ system is pH-dependent and generally performs better in alkaline media^34^,^35^. Therefore, the effect of pH value was firstly investigated over the range of 9.8–10.8. As shown in Fig. 5A, the maximum enhancement of ECL intensity was observed at pH 10.4. The reason might be as follows: when pH value increased from 9.8 to 10.4, the oxidation strength of H_2_O_2_ was enhanced and the ECL intensity increased gradually. After pH value above 10.4, highly alkaline surrounding facilitated the decomposition of H_2_O_2_ leading to the formation of bubbles^36^. So that pH 10.4 was selected as the optimal condition.

The concentration of co-reactant H_2_O_2_ in the buffer was very crucial to the proposed immunosensor. As shown in Fig. 5B, the ECL intensity increased with the concentration of H_2_O_2_ from 5 to 25 mM due to the co-oxidation function of H_2_O_2_. When the concentration of H_2_O_2_ was above 25 mM, ECL intensity weakened due to the decrease of the ion index of the substrate solution. Thus, 25 mM was chosen as the optimal concentration of H_2_O_2_.

To obtain an optimal ECL signal, the amount of Au NPs on the surface of PPy was seclected. In this experiment, other conditions were fixed and different adding volume of Au NPs (0, 0.2, 0.4, 0.6, 0.8, 1.0, 1.2 and 1.4 mL) was added. The result was shown in Figure S1. When the adding volume of Au NPs exceeded 1.0 mL, the ECL intensity maintained a stable value, meaning Au NPs reached the saturation adsorption. So 1.0 mL was selected as the optimal adding volume of Au NPs.

### Analytical performance of the immunosensor toward target CEA

Under the optimized experimental conditions, the developed label-free immunosensor was applied to quantify the target CEA from 0.01 pg/mL to 10 ng/mL. As seen from Fig. 6A, a linear proportion between the ECL intensity and the logarithmic values of the CEA concentration could be achieved in the dynamic range from 0.01 pg/mL to 10 ng/mL. The linear equation was *I* = 6293.25 − 1854.14 lg (*c*, ng/mL) with a correlation coefficient of 0.9945. Compared with limit of detection (LOD) for amperometry (60 pg/mL)^8^, voltammetry (5 pg/mL)^37^, potentiometry (500 pg/mL)^38^, capillary electrophoresis (4.8 pg/mL)^9^, fluorometry (5 pg/mL)^39^, electrochemistry (2.36 pg/mL)^11^ (Table S1), the proposed method exhibited a lower LOD (3 fg/mL, signal/noise [S/N] ratio = 3). This is probably due to the enhancement of Au NPs and the excellent conductivity of Au NPs@PPy nanocomposites. Based on the above facts, it could be speculated that the designed immunoassay was effective for CEA detection.

The repeatability of the proposed immunosensor was studied (Fig. 6B). A series of five electrodes were prepared for the detection of 1 ng/mL CEA determined in 10 mL of 0.067 mM CBS (pH 10.4) containing 25 mM H_2_O_2_. And the relative standard deviation (RSD) was 3.90%, which indicated an acceptable level of the repeatability for the immunosensor.

The specificity is of great concern for the practical use of the biosensor. The assay was measured in the presence of two kinds of potential interferents and one kind of pure no-specific antigens, including BSA (10 ng/mL), glucose (10 ng/mL) and α-Fetoprotein (10 ng/mL), respectively. It was seen from Fig. 6C, the ECL signal of the established sensor treated with the mixture of interfering substances and CEA antigen (0.1 ng/mL) suffered from negligible affection comparing with the 0.1 ng/mL CEA. The corresponding RSD of 3.07% was considered to be the acceptable range, which manifested the proposed immunosensor had an excellent specificity for CEA detection.

Additionally, the ECL stability of developed immunosensor modified with 0.1 ng/mL was investigated under consecutively giving potential to the modified electrode 20 times. A relatively stable ECL emission curve (Fig. 6D) was obtained with the RSD of 0.71%. The great stability derived from the following reasons: firstly, anti-CEA and luminol could be strongly coated on the surface of Au NPs@PPy. Secondly, the consecutive reaction between luminol and H_2_O_2_ was limited by single-step cycle pulse which would keep the ECL intensity of luminol. Thus, the stability of as-prepared ECL immunosensor was quite well for CEA detection.

### Application

As further proof of the good performance of the introduced method, the practical application of the label-free ECL immunosensor was investigated by analysis of human serum samples. Firstly, the human blood was pretreated by centrifugation to remove the blood cells and other blood sediment. Then, the human serum was taken out and the samples were diluted with pH 7.4 PBS until a level that was during the calibration range. After the prepared samples were determined, different concentration (1.00, 3.00, 5.00 ng/mL) of standard CEA solution was added to human serum samples by standard addition methods. The result was shown in Table S2, it demonstrated that the RSD and recoveries were in the range of 1.59–4.50% and 97.4–101% respectively. So the as-prepared immunosensor performed a satisfactory result and it might be preliminarily applied for the determination of CEA in real samples.

## Conclusion

In this study, a label-free ECL immunosensor has been developed based on the luminol-Au NPs@PPy as antibody carriers and sensing platform, which provides the advantages of simplicity in design and in operation from an application standpoint. PPy could load a large amount of Au NPs to enhance ECL response of luminol/H_2_O_2_ and Au NPs improved the absorption capacity of antibody. The ECL immunosensor exhibited excellent accuracy, precision and sensitivity. It also displayed a linear response with a wide range and a low detection limit for quantitative detection of AFP. The employment of single-step cycle pulse could control the consecutive reaction between luminol and H_2_O_2_ efficiently, which also economized reagents and exhibited good operational stability. This proposed method would have potential application in clinical monitoring of CEA.

## Materials and Methods

### Apparatus

The ECL emission measurements were performed on a MPI-F flow-injection chemiluminescence detector (Xi’an Remax Electronic Science Technology, China) and electrochemical measurements were carried out on a CHI760D electrochemical workstation (Chenhua Instrument Shanghai, China) by using a three-electrode system consisted of a platinum wire as auxiliary electrode, an Ag/AgCl electrode as reference electrode and the prepared electrodes with different CEA concentrations as working electrode. Scanning electron microscope (SEM) images were obtained using a field emission SEM (Zeiss, Germany). Electrochemical impedance spectroscopy (EIS) measurements were performed using IM6e Electrochemical Interface (Zahner, Germany). UV-vis spectra were carried out by using a Lambda 35 UV-vis spectrometer (PerkinElmer, United States). Fourier transform infrared spectroscopy (FTIR) spectrum was recorded on a VERTEX 70 spectrometer (Bruker, Germany).

### Materials

CEA and antibody of CEA were purchased from Wang Er Biochemical Reagents (Beijing, China). Luminol and Bovine serum albumin (BSA, 96–99%) were obtained from Sigma-Aldrich (Beijing, China). HAuCl_4_·6 H_2_O was purchased from Alfa Aesar. Pyrrole was gained from Sinopharm chemical reagent Co., Ltd. All other chemicals were of analytical grade and used without further purication. Phosphate buffered saline (PBS) was prepared by using 0.067 mM Na_2_HPO_4_ and 0.067 mM KH_2_PO_4_. Carbonate buffered saline (CBS) was obtained by using 0.067 mM Na_2_CO_3_ and 0.067 mM NaHCO_3_. Ultrapure water (18.25 MΩ cm, 25 °C) was used for the experiment.

### Preparation of polypyrrole

PPy was prepared by chemical oxidation polymerization according to the literature^25^. Firstly, the pyrrole monomer was purified by pretreatment. Then, the processed pyrrol (0.2 M) was dissolved in 50 mL mixture of water and ethanol (1:1). Then, 50 mL of ferric chloride (0.1 M) was added dropwise. The mixture was kept for 24 h with continuous stirring at room temperature, followed by filtration and washing several times with water and ethanol mixture to remove excess ferric chloride. Finally, the obtained black polymer was dried in vacuum oven at 60 °C for 24 h.

### Preparation of luminol-Au NPs@PPy composites

Luminol-Au NPs@PPy nanocomposites were prepared by the following steps. In the first, Au NPs were synthesized as described previously^40^ with a slightly modification. Then, 10 mL of prepared Au NPs solution was added into 10 mL of PPy dispersion containing 20 mg of PPy. The mixture was under continuous ultrasound for 30 min and stirred for 24 h. Following centrifugation, the resulting Au NPs@PPy was obtained and dispersed in 10 mL of ultrapure water.

Next, 2 mL of Au NPs@PPy dispersion (2 mg/mL) and 2 mL of luminol (1 mM) were mixed. The reaction was allowed to continue for 12 h with vigorous shaking at room temperature under lucifugal situation, followed by centrifugation at 8000 rpm for 10 min to remove unloaded luminol. Finally, the luminol-Au NPs@PPy composites were dispersed in 2 mL of ultrapure water and stored at 4 °C.

### Immobilization of anti-CEA on to the luminol-Au NPs@PPy

**Figure 1:**
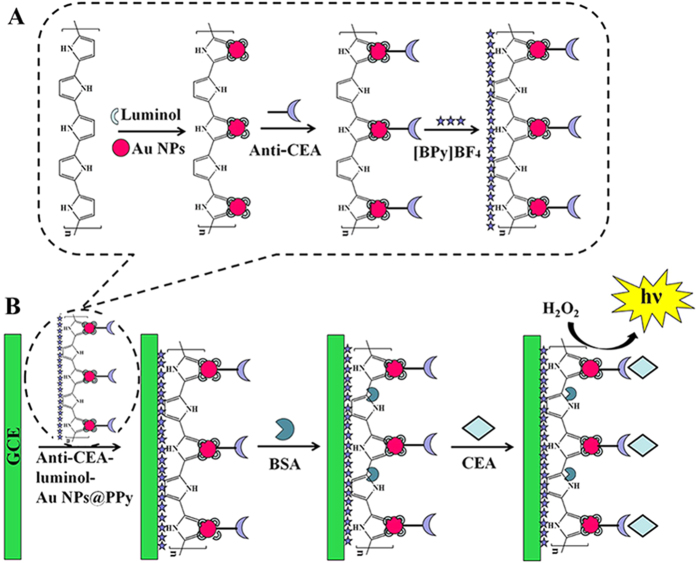
(**A**) The preparation procedure of the anti-CEA-luminol-Au NPs@PPy. (**B**) The schematic illustration of the label-free electrochemiluminescence immunosensor.

**Figure 2:**
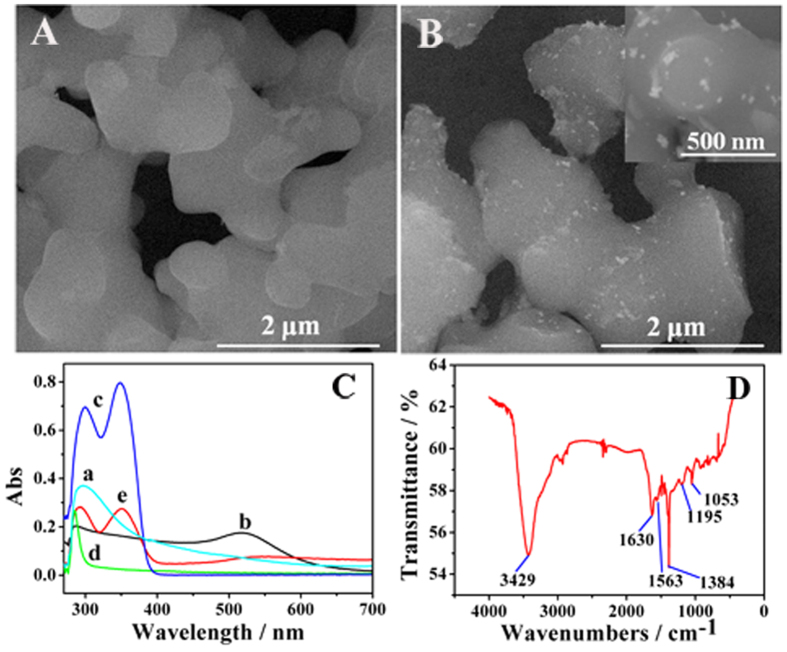
SEM images of PPy (**A**) and Au NPs@PPy (**B), (C**) UV-vis spectrums of PPy (a), Au NPs (b), luminol (c), anti-CEA (d), anti-CEA-luminol-Au NPs@PPy (e). (**D**) The FT-IR characterization of PPy.

**Figure 3:**
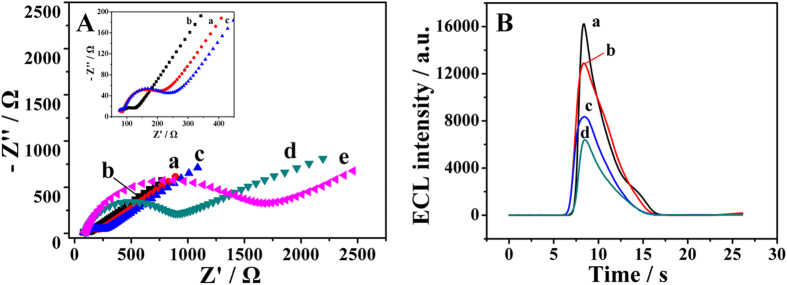
(**A**) EIS of the electrode at different modification stages: (a) bare GCE, (b) luminol-Au NPs@PPy/GCE, (c) anti-CEA-luminol-Au NPs@PPy/GCE, (d) BSA/anti-CEA-luminol-Au NPs@PPy/GCE and (e) CEA/BSA/anti-CEA-luminol-Au NPs@PPy/GCE measured in ferricyanide solutions (Fe(CN)_6_^3−/4−^, 2.5 mM, pH 7.4). (**B**) ECL intensity profiles of the luminol-Au NPs@PPy/GCE (curve a), anti-CEA-luminol-Au NPs@PPy/GCE (curve b), BSA/anti-CEA-luminol-Au NPs@PPy/GCE (curve c) and CEA/BSA/anti-CEA-luminol-Au NPs@PPy/GCE (curve d) measured in CBS (pH 10.4) containing 25 mM H_2_O_2_.

**Figure 4:**
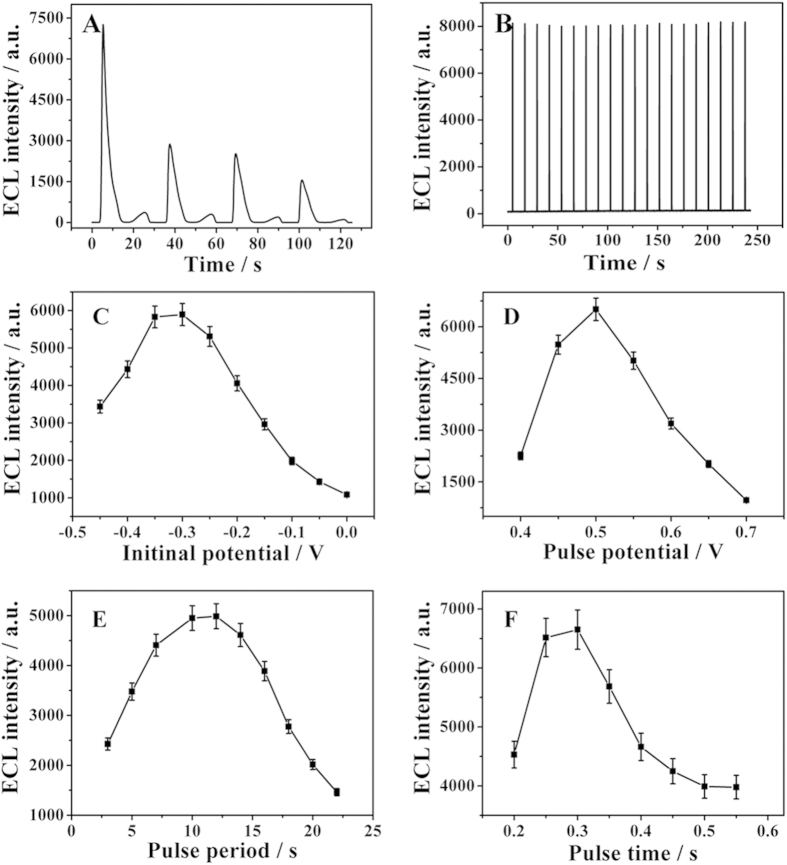
ECL intensity of immunosensor with different electrochemical techniques including cyclic voltammetry (**A**) and single-step cycle pulse (**B**).The effect of initial potential (**C**), pulse potential (**D**), pulse period (**E**) and pulse time (**F**). Error bar = SD (n = 3).

**Figure 5:**
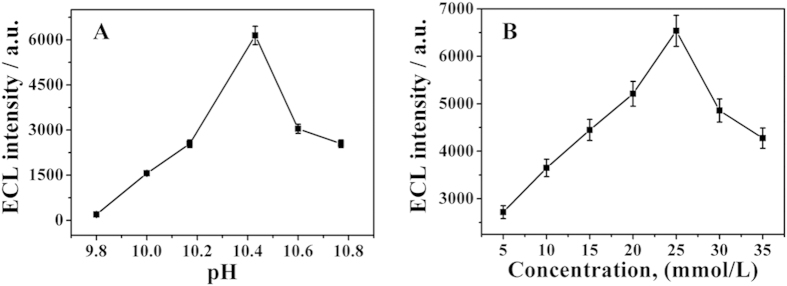
The effect of pH (**A**) and concentration of H_2_O_2_ (**B**). Error bar = SD (n = 3).

**Figure 6:**
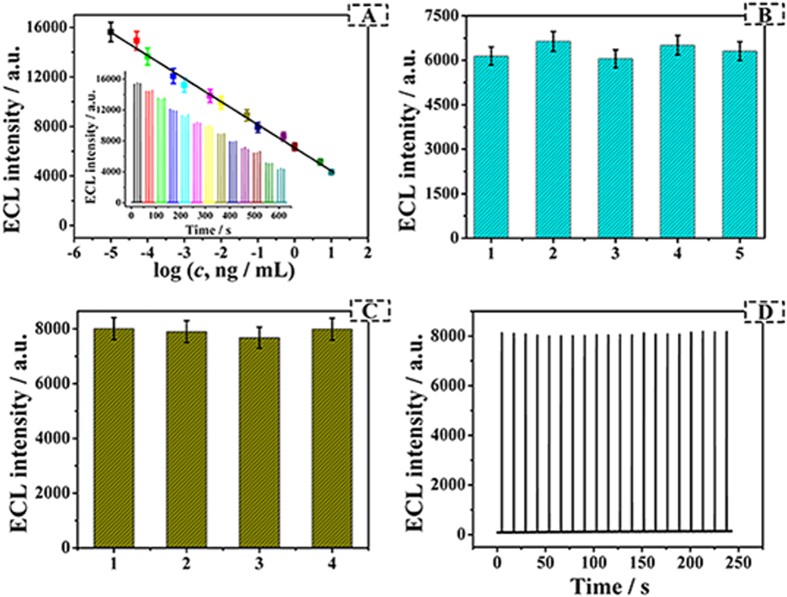
(**A**) Calibration curve of the immunosensor for different concentrations of CEA measured in CBS (pH 10.4) containing 25 mM H_2_O_2_ (insert is the ECL response of the immunosensor to different concentrations of CEA, from left to right: 0.00001, 0.00005, 0.0001, 0.0005, 0.001, 0.005, 0.01, 0.05, 0.1, 0.5, 1, 5 and 10 ng/mL). (**B**) The repeatability of the proposed ECL modified electrodes with 1 ng/mL CEA. (**C**) ECL intensity of the ECL sensor to 0.1 ng/mL CEA (1), 0.1 ng/mL CEA + 10 ng/mL BSA (2), 0.1 ng/mL CEA + 10 ng/mL glucose (3), 0.1 ng/mL CEA + 10 ng/ mL α-Fetoprotein (4), Error bar = SD (n = 3). (**D**) The stability of ECL modified electrode with 0.1 ng/mL CEA measured in CBS (pH 10.4) containing 25 mM H_2_O_2_ under single-step cycle pulse.

The fabrication process of anti-CEA-luminol-Au NPs@PPy was shown in Fig. 1A. 0.1 mL of anti-CEA (10 μg/mL) was added into 1 mL of as prepared luminol-Au NPs@PPy solution, followed by incubating for 24 h at 4 °C. After that, an excess of anti-CEA was removed by centrifugation. At last, the anti-CEA-luminol-Au NPs@PPy composites were dispersed in 1 mL of [BPy]BF_4_ ionic liquid (50 mg/mL) and stored at 4 °C for further use.

### Fabrication of the label-free ECL immunosensor

Figure 1B showed the fabrication procedure of the immunosensor. A glass carbon electrode (GCE) with 4 mm diameter was firstly polished repeatedly with alumina in polishing cloth, followed by rinsing thoroughly with ultrapure water prior to use. First, a solution of anti-CEA-luminol-Au NPs@PPy (2 mg/mL, 6 μL) was added onto the cleaned electrode. After dried, 3 μL of BSA (1 wt%) was dropped on the anti-CEA-luminol-Au NPs@PPy to eliminate nonspecific binding spots and dried again. Following a thorough rinse with PBS (pH 7.4), the electrode was incubated with a series of CEA solutions (6 μL) with different concentrations. After thoroughly washing, the modified electrode was stored at 4 °C for further measurement.

### Experimental measurements

The ECL measurements of the prepared electrodes above were performed in an ECL detector cell containing 10 mL of CBS (pH 10.4, 0.067 M) and 25 mM H_2_O_2_. The ECL intensity was obtained when single-step cycle pulse was enforced to the electrode with initial potential of −0.3 V, pulse potential of 0.5 V, pulse period of 12 s and pulse time of 0.3 s. Meanwhile, the voltage of the photomultiplier tube (PMT) was set at 700 V with 0.1 V/s scan rate in the process of CEA detection. Then, the modified electrodes were placed in the ECL cell and measured the ECL signal.

## Additional Information

**How to cite this article**: Zhu, W. *et al*. Single-step cycle pulse operation of the label-free electrochemiluminescence immunosensor based on branched polypyrrole for carcinoembryonic antigen detection. *Sci. Rep.* **6**, 24599; doi: 10.1038/srep24599 (2016).

## Supplementary Material

Supplementary Information
