# Supplementary material for: Single-step cycle pulse operation of the label-free electrochemiluminescence immunosensor based on branched polypyrrole for carcinoembryonic antigen detection
Source: Sci Rep. 2016 Apr 19;6:24599. doi: 10.1038/srep24599 (PMC4835776; doi:10.1038/srep24599)
Supplement: Supplementary Information [file srep24599-s1.pdf]

Supporting Information

Single-step cycle pulse operation of the label-free electrochemiluminescence immunosensor based on branched polypyrrole for carcinoembryonic antigen detection

Wenjuan Zhu[a], Qi Wang[b], Hongmin Ma[a], Xiaohui Lv[a], Dan Wu[a], Xu Sun[a], Bin Du[a], Qin Wei[a]*

[a]*Key Laboratory of Chemical Sensing & Analysis in Universities of Shandong, School of Chemistry and Chemical Engineering, University of Jinan, Jinan 250022, P.R. China*

[b]*School of Material Science and Engineering, University of Jinan, Jinan 250022, P.R. China*

***Corresponding author.**
Tel: +86 531 82767872

Fax: +86 531 82767367

E-mail address: sdjndxwq@163.com (Q. Wei)

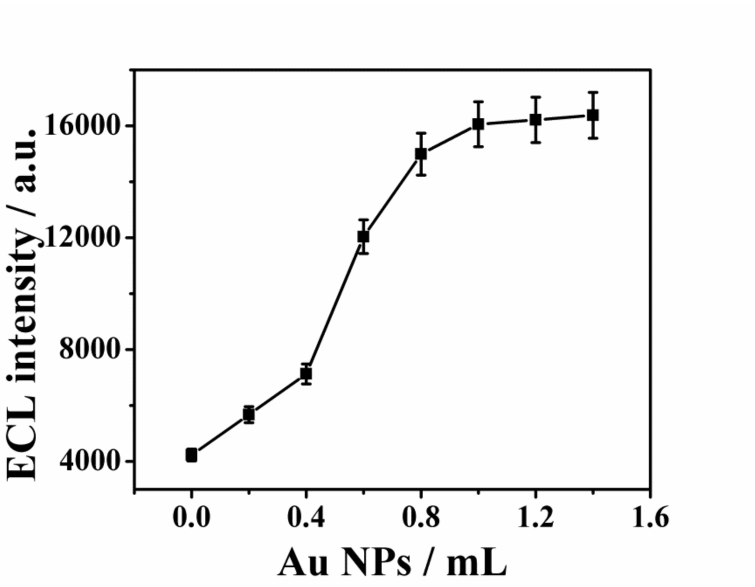

Figure S1. The effect of the amount of Au NPs. Error bar = SD (n = 3).

Table S1. A comparison of the performance of the proposed and referenced sensors for CEA.

Method	Material of sensor	Linear range (ng/mL)	Detection limit (pg/mL)	References
Amperometry	Nano-Au/chitosan composite	0.2-120	60	[8]
Voltammetry	Multiarmed star-like Pt nanowires	0.01-60	5	[37]
Potentiometry	Functionalized core/shell Fe_3O_4@Ag NPs	1.5-200	500	[38]
Capillary electrophoresis	HRP-aptamer/GO	0.0654-6.54	4.8	[9]
Fluorometry	GO/QD-aptamer	0.257-12.9	5	[39]
Electrochemistry	Hyperbranched polyester	0.08-80	2.36	[11]
Electrochemiluminescence	Polypyrrole loaded AuNPs	0.00001-10	0.003	This work

Table S2. The recoveries of CEA determination in human serum samples using the proposed ECL modified electrodes measured in 10 mL CBS (pH 10.4) containing 25 mM H_2O_2 (n = 5).

Samples（ng/mL）	Addition（ng/mL）	Detection（ng/mL）	Average value（ng/mL）	RSD（%）	Recovery（%）
	1.00	1.42, 1.54, 1.43, 1.47, 1.57	1.49	4.50	97.4
0.530	3.00	3.42, 3.38, 3.57, 3.62, 3.54	3.51	2.73	99.4
	5.00	5.57, 5.65, 5.47, 5.69, 5.51	5.58	1.59	101
